# Transient Global Amnesia (TGA): Influence of Acute Hypertension in Patients Not Adapted to Chronic Hypertension

**DOI:** 10.3389/fneur.2021.666632

**Published:** 2021-07-08

**Authors:** Andreas Rogalewski, Anne Beyer, Anja Friedrich, Jorge Plümer, Frédéric Zuhorn, Isabell Greeve, Randolf Klingebiel, Friedrich G. Woermann, Christian G. Bien, Wolf-Rüdiger Schäbitz

**Affiliations:** ^1^Department of Neurology, Evangelisches Klinikum Bethel, University Hospital OWL of the University Bielefeld, Bielefeld, Germany; ^2^Department of Psychology, Bielefeld University, Bielefeld, Germany; ^3^Department of Neuroradiology, Evangelisches Klinikum Bethel, University Hospital OWL of the University Bielefeld, Bielefeld, Germany; ^4^Department of Epileptology (Krankenhaus Mara), Bielefeld University, Medical School, Bielefeld, Germany

**Keywords:** transient global amnesia, stroke, risk factors, hypertensive crisis, diffusion-weighted MRI, hypertension

## Abstract

**Objective:** Transient global amnesia (TGA) is defined by an acute memory disturbance of unclear etiology for a period of <24 h. Several studies showed differences in vascular risk factors between TGA compared to transient ischemic attack (TIA) or healthy controls with varying results. This retrospective and cross-sectional study compares the cardiovascular risk profile of TGA patients with that of acute stroke patients.

**Methods:** Cardiovascular risk profile and MR imaging of 277 TGA patients was retrospectively analyzed and compared to 216 acute ischemic stroke patients (26% TIA).

**Results:** TGA patients were significantly younger and predominantly female compared to stroke patients. A total of 90.6% of TGA patients underwent MRI, and 53% of those showed hippocampal diffusion-weighted imaging (DWI) lesions. Scores for cerebral microangiopathy were lower in TGA patients compared to stroke patients. After statistical correction for age, TGA patients had higher systolic and diastolic blood pressure, higher cholesterol levels, lower HbA1c, as well as blood glucose levels, and lower CHA_2_DS_2_-VASc scores. Stroke patients initially displayed higher CRP levels than TIA and TGA patients. TGA patients without DWI lesions were older and showed higher CHA_2_DS_2_-VASc scores compared to TGA patients with DWI lesions.

**Conclusion:** This study revealed significant differences between TGA and stroke patients in regard to the cardiovascular risk profile. Our main findings show a strong association between acute hypertensive peaks and TGA in patients not adapted to chronic hypertension, indicating a vascular cause of the disease.

## Introduction

Transient global amnesia (TGA) is an acute disturbance of episodic memory for a period of <24 h that usually occurs in middle-aged and elderly individuals. During this phase, anterograde amnesia, and aspects of retrograde amnesia exist without further cognitive impairment or focal neurologic symptoms. The syndrome was first described in 1956 (1). Various aetiologic factors such as spreading-depression caused by migraine, epilepsy, cerebral ischemia, and venous flow anomalies have been discussed as potential cause of the disease (2), but the exact pathophysiological mechanism remains unknown. The annual TGA incidence ranges from 3.4 to 10.4 per 100.000 increasing to 32 per 100.000 in individuals above the age of 50 (3, 4).

Based on the clinical syndrome, an affection of the mediobasal temporal lobes including the hippocampi was assumed, since these structures are involved in both memory consolidation and memory recall (5). Studies using diffusion-weighted imaging (DWI) confirmed this hypothesis by demonstrating hippocampal DWI lesions in more than half of the patients with an acute TGA in the time window up to 48 h after symptom onset (6–11). Such focal, punctate diffusion lesions are detected both unilaterally (in both hemispheres) as well as bilaterally. Interestingly, TGA-patients with and without DWI lesions do not differ in clinical symptoms and cognitive functions in the long term (12). Although, no permanent lesions could be shown by MRI (8, 12), electrophysiological abnormalities in event-related potentials could prove prolonged effects even after 17 months (13). These observations may suggest permanent functional damage within the hippocampal structures and its neuronal networks, although, confirmation of this long-term memory impairment has not yet been published.

Due to the well-described occurrence of DWI lesions within the first days following TGA onset, a vascular genesis of the syndrome was intensively discussed. Several studies reveal neither a conspicuous association to cardiovascular risk factors nor an accumulation of previous or future cerebral infarctions in TGA-patients (14–17), whereas other studies reported differences in vascular risk factors between TGA patients and TIA patients or normal controls (15, 18–22).

Therefore, the aim of our study was to assess clinical characteristics, neuroimaging findings, and comorbidities in TGA-patients with a focus on cardiovascular risk factors.

## Methods

### Patients

We conducted a cross-sectional study *via* retrospective analysis of patient records. A total of 277 patients diagnosed with TGA, according to the criteria defined by Hodges and Warlow (1990) (23), from one German hospital were recruited between January 1, 2013, and December 31, 2019. As a reference condition, 216 consecutive patients of our Stroke Unit with acute ischemic stroke or transient ischemic attack were included; these data were collected between November 24, 2018, and June 3, 2019.

### Procedure

Patient characteristics were evaluated including demographics, cardiovascular risk factors, and imaging findings (MRI or CT scan, ultrasound, echocardiography).

MRI scans were evaluated to disclose stroke and to detect hippocampal DWI lesions. The extent of cerebral microangiopathy was assessed using Fazekas' score (0–3). The presence of cerebrovascular stenosis was assessed using ultrasound. Blood pressure on admission and laboratory parameters (cholesterol level, glucose level, HbA1c, CRP) were obtained from emergency room records. Echocardiography was evaluated for left ventricular ejection fraction and septal hypertrophy. Septal hypertrophy as a possible indicator for chronic hypertension has been defined as the presence of increased septal thickness (women > 9 mm, men > 10 mm) (24). In addition, CHA_2_DS_2_-VASc scores were determined for all patients at the time of current admission. This means that in the group of stroke patients, the current stroke was not yet included, but a previous stroke event was considered.

## Acute Hypertension vs. Chronic Hypertension/Hypertensive Crisis

Blood pressure values on admission were recorded and evaluated as single blood pressure values. Chronic hypertension was assumed if antihypertensive medication was required during the hospital stay and at discharge.

In general, hypertensive crises are defined as an increase in systolic blood pressure >179 mm Hg or diastolic blood pressure >109 mm Hg (25). There is a strong association with the occurrence of end-organ damage, with the brain being an elective and early target. In this context, hypertensive encephalopathy represents typical hypertensive sequelae of brain damage. It is known that previously normotensive individuals can develop signs of encephalopathy as a result of failure of the upper limit of cerebral vascular autoregulation (autoregulation breakthrough) at blood pressures as low as 160/100 mm Hg, whereas individuals with chronic hypertension may not do so until the blood pressure rises to 220/110 mm Hg or greater (26). Therefore, we defined hypertensive crisis on admission as the following: (1) in chronic hypertensive patients (= antihypertensive medication was required during hospital stay and at discharge), systolic blood pressure value > 179 mm Hg and/or diastolic blood pressure value > 109 mm Hg, and (2) in normotensive patients (no antihypertensive medication was required after day of admission and at discharge), systolic blood pressure value > 159 mm Hg, and/or diastolic blood pressure value > 99 mm Hg.

### Data Analyses

Statistical analysis of the data was carried out using the Statistical Package for the Social Sciences (SPSS) version 25 (IBM, 2018). Descriptive statistics were displayed as mean ± standard deviation for continuous data and frequencies with percentages for categorical variables. Normal distribution was assessed *via* Shapiro-Wilk test with *p* < 0.05 indicating non-normal distribution, and homoscedasticity was assessed visually *via* q-q-plots.

**Figure 1:**
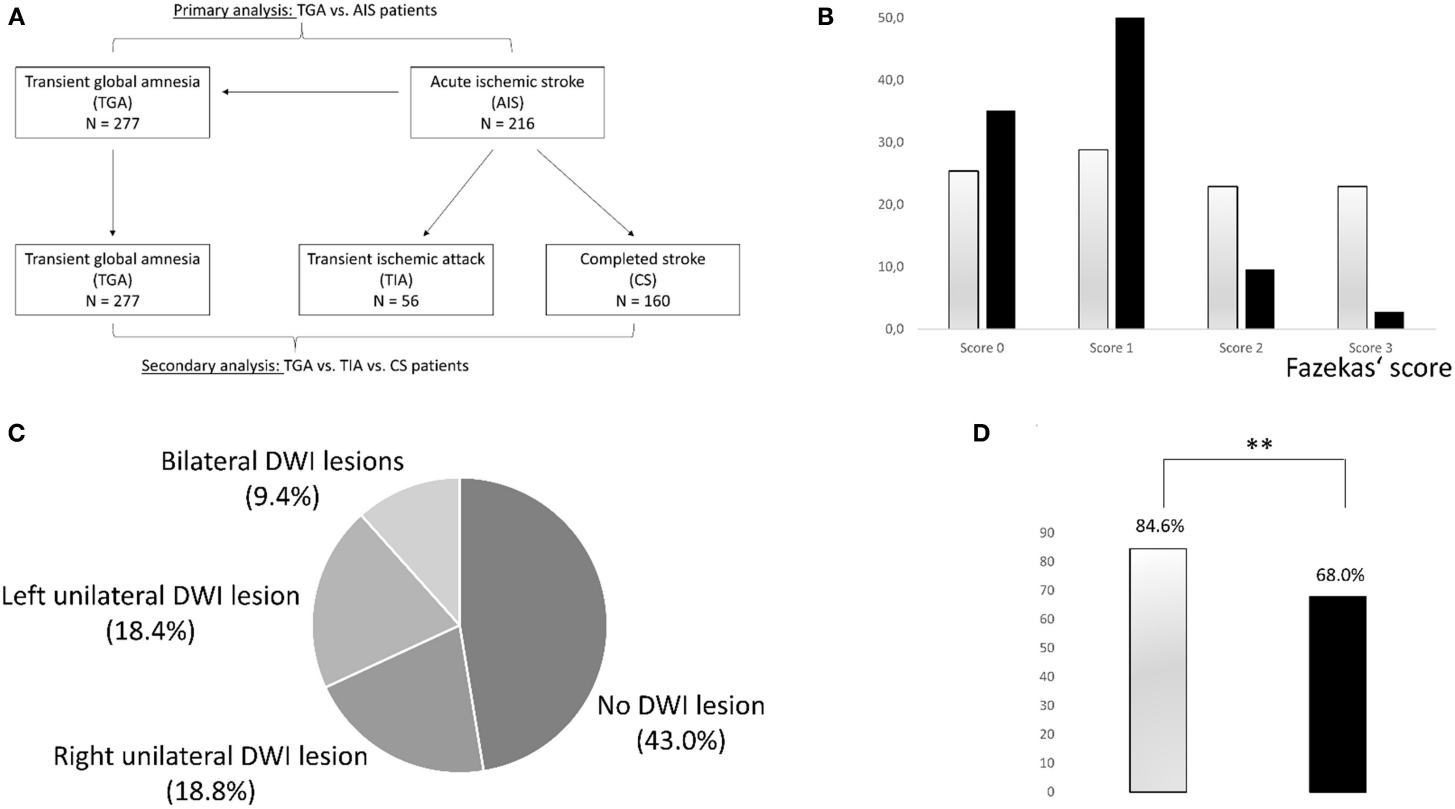
**(A)** Presentation of patient groups and analyses. **(B)** Severity distribution of cerebral microangiopathy using Fazekas' score in patients with stroke (gray) and TGA (black). Patients with TGA are more likely to have no or mild microangiopathy, while moderate or severe microangiopathy is more common in stroke patients. **(C)** Distribution of hippocampal DWI lesions in patients with TGA. **(D)** Presence of septal hypertrophy in transthoracic echocardiography as a potential sign of chronic hypertension in patients with AIS (gray bar) vs. TGA patients (black bar). Patients with AIS displayed septal hypertrophy more frequently (^**^p < 0.001).

The profile of cardiovascular risk factors was compared between TGA-patients and patients with acute ischemic stroke (AIS-patients) with parametric *t*-tests or non-parametric Mann-Whitney *U*-tests, depending on normal distribution. In these comparisons, patients with completed stroke (CS-patients) and transient ischemic attack (TIA-patients) were analyzed together as AIS-patients without differentiating between these two groups (Figure 1A). Secondary analyses were conducted between TGA-patients, CS-patients, and TIA-patients using ANOVAs or non-parametric Kruskal-Wallis-tests. *Post-hoc* tests were conducted using parametric *t*-tests (ANOVA) or non-parametric Mann-Whitney *U*-tests (Kruskal-Wallis tests). *Post-hoc p*-values were adjusted *via* inverse Bonferroni (*p*_adj_ = *p* ^*^ k, k being the number of comparisons) in order to correct for multiple testing and alpha error accumulation.

Data displayed demographic differences between the three conditions; therefore, exploratory analyses separated by age and gender were carried out to correct this influence. More sophisticated approaches, e.g., *via* MANCOVAs or (logistic) regression analyses, were not feasible due to skewed data that led to severe requirement violations. Additional MRI and hippocampal lesion comparisons were conducted using parametric *t*-tests and non-parametric Mann-Whitney-U and Kruskal-Wallis tests.

Effect sizes were calculated as Cohen's r (r = z/√N) with r >.10 as a small effect, r >.30 as a moderate effect, and r >.50 as a large effect. Missing data were excluded pairwise from the analyses.

## Results

### Primary Analysis: TGA vs. AIS Patients

#### Demographic Characteristics

In total, 277 TGA-patients and 216 AIS-patients were analyzed in this study. Mean age was 67.1 ± 10.3 years in TGA-patients and 70.6 ± 14.9 years in AIS-patients. TGA-patients were significantly younger than AIS-patients. In addition, statistically significant differences were observed for gender, with a higher percentage of women in TGA-patients than in AIS-patients (TGA: 61%, AIS: 44%). Further demographic characteristics are presented in Tables 1, 2.

**Table 1:** Baseline characteristics of TGA patients (*N* = 277).

Age	67.1 ± 10.3 years [20; 90]
Male	39%
**Presence/side of DWI lesion**	
No lesion	119 (43.0%)
Left hippocampus	51 (18.4%)
Right hippocampus	52 (18.8%)
Bilateral hippocampus	29 (10.5%)
No MRI scan	26 (9.4%)
**Risk factors**	
Hypertension	200 (73.3%)
Systolic pressure at admission	170 ± 23 mm Hg
Diastolic pressure at admission	92 ± 13 mm Hg
Diabetes mellitus	13 (4.7%)
Serum glucose level at admission	117.3 ± 20.9 mg/dl
HbA1c level at admission	5.5 ± 0.6%
Hypercholesterolemia (Serum cholesterol > 200 mg/dl)	88/248 (35.5%)
Serum cholesterol level at admission	216.9 ± 41.7 mg/dl
Atrial fibrillation	19 (6.9%)
CHA_2_DS_2_-VASc score	2.9 ± 1.6 [0;7]
Former stroke	38 (13.7%)
Cerebral stenosis	18/266 (6.8%)
Inflammation	
CRP level at admission	2.1 ± 3.4 mg/l
LVEF < 50%	3/135 (2.2%)
Septal hypertrophy (women > 9 mm, men > 10 mm)	87/128 (68.0%)
**Cerebral microangiopathy (MRI or CT scan)**	
No microangiopathy	98 (35.4%)
Mild (Fazekas' score 1)	138 (49.8%)
Moderate (Fazekas' score 2)	26 (9.4%)
Severe (Fazekas' score 3)	12 (4.3%)
No image	3 (1.1%)
**Treatment at discharge**	
Antiplatelet therapy	170 (61.4%)
Oral anticoagulation	19 (6.9%)
Statin therapy	160 (57.8%)
Antihypertensive therapy	201 (72.6%)

**Table 2:** Comparison of TGA patient characteristics with AIS patients (columns 2–4) and comparison of TGA patient characteristics with CS-patients and TIA-patients (columns 5–9).

	**TGA patients (*N* = 277)**	**AIS patients (*N* = 216)**	**Comparisons** **TGA vs. AIS**	**TGA patients (*N* = 277)**	**CS patients (*N* = 160)**	**TIA patients** **(*N* = 56)**	**Comparisons TGA vs. CS vs. TIA**	***Post-hoc* tests** **TGA vs. CS vs. TIA**
Age	67.1 ± 10.3 277/277	70.3 ± 14.8216/216	U = 35,904.500, z = 3.817, *p* < 0.001^b^	67.1 ± 10.3277/277	71.1 ± 13.9 160/160	69.4 ± 14.756/56	χ^2^ 16.169, *p* < 0.001, df = 2^d^	TGA < CS (z = −4.002, *p* < 0.001)^e^
Male	39.0% 108/277	56.0%121/216	χ^2^ = 14.149, *p* < 0.001^a^	39.0%108/277	55.0% 88/160	58.9%33/56	χ^2^ = 14.407, *p* = 0.001, df = 2^d^	TGA (z = −3.762, *p* < 0.001)^e^
CHA_2_DS_2_-VASc score on admission^e^	2.9 ± 1.6 277/277	3.7 ± 1.7216/216	U = 21,823.000, z = 5.241, *p* < 0.001^b^	2.9 ± 1.6277/277	3.7 ± 1.7 160/160	3.5 ± 1.756/56	χ^2^ 27.620, *p* < 0.001, df = 2^d^	CS > TGA (z = −4.947, *p* < 0.001)^e^ TIA > TGA (z = 2.946, *p* = 0.010)^e^
Diabetes mellitus	4.7% 13/276	20.4%44/216	χ^2^ = 29.011, *p* < 0.001^a^	4.7%13/276	20.0% 32/160	21.4%12/56	χ^2^ = 29.093, df = 2, *p* < 0.001^b^	TGA (z = −5.386, *p* < 0.001)^e^ CS (z = 4.049, *p* < 0.001)^e^
Antihypertensive therapy at discharge	73.3% 200/273	90.7%196/216	χ^2^ = 23.926, *p* < 0.001^a^	73.3%200/273	91.3% 146/160	89.3%50/56	χ^2^ = 25.676, df = 2, *p* < 0.001^b^	TGA (z = −4.891, *p* < 0.001)^e^ CS (z = 4.035, *p* < 0.001)^e^
LVEF < 50%	2.2% 3/135	5.3%10/190	χ^2^ = 1.901, *p* = 0.168^a^	2.2%3/135	7.1%10/140	0%0/50	χ^2^ = 6.796, df = 2, *p* = 0.033^a^	n.s.
Septal hypertrophy (women > 9 mm, men > 10 mm)	68.0% 87/128	84.6%159/188	χ^2^ = 12.177, *p* < 0.001^a^	68.0%87/128	87.7% 121/138	76.0%38/50	χ^2^ = 15.081, df = 2, *p* = 0.001^a^	TGA (z = 3.490, *p* = 0.002)^d^ CS (z = −3.706, *p* = 0.001)^d^
Cerebral stenosis	6.8% 18/266	19.0%41/216	χ^2^ = 16.556, *p* < 0.001^a^	6.8%18/266	23.1% 37/160	7.1%4/56	χ^2^ = 26.419, df = 2, *p* < 0.001^a^	TGA (z = −4.069, *p* < 0.001)^d^ CS (z = 5.139, *p* = 0.004)^d^
Atrial fibrillation	6.9% 19/275	18.1%39/216	χ^2^ = 14.429, *p* < 0.001^a^	6.9%19/275	19.4% 31/160	14.3%8/56	χ^2^ = 15.460, df = 2, *p* < 0.001^a^	TGA (z = −3.799, *p* < 0.001)^d^ CS (z = 3.610, *p* = 0.004)^d^
Cholesterol serum level on admission	216.9 ± 41.7 248/277	191.9 ± 48.1214/216	U = 17,355.000, z = −3.837, *p* < 0.001^b^	216.9 ± 41.7248/277	191.7 ± 48.1 158/160	192.6 ± 48.456/56	χ^2^ 37.624, *p* < 0.001, df = 2^c^	TGA > TIA (z = −3.573, *p* = 0.001)^d^ TGA > CS (z = 5.760, *p* < 0.001)^d^
Serum glucose level on admission	117.3 ± 20.9 269/277	134.1 ± 47.6213/216	U = 34,350.500, z = 3,755, *p* < 0.001^b^	117.3 ± 20.9269/277	132.6 ± 44.4 158/160	138.5 ± 56.155/56	χ^2^ 14.233, *p* = 0.001, df = 2^c^	TGA < TIA (z = 2.611, *p* = 0.027)^d^ TGA < CS (z = −3.291, *p* = 0.003)^d^
HbA1c on admission	5.49 ± 0.62 243/277	6.00 ± 1.13212/216	U = 32,179.000, z = 4.601, *p* < 0.001^b^	5.49 ± 0.62243/277	6.00 ± 1.08156/160	5.99 ± 1.2556/56	χ^2^ 22.968, *p* < 0.001, df = 2^c^	CS > TGA (z = −4.753, *p* < 0.001)^d^
CRP on admission	2.1 ± 3.4 275/277	11.1 ± 24.7216/216	U = 38,497.000, z = 5.641, *p* < 0.001^b^	2.1 ± 3.4275/277	13.1 ± 27.5 160/160	5.4 ± 12.556/56	χ^2^ 42.826, *p* < 0.001, df = 2^c^	CS > TGA (z = −6.501, *p* < 0.001)^d^ CS > TIA (z = −3.318, *p* = 0.003)^d^
Systolic blood pressure (SBP) on admission	170.0 ± 23.3 237/277	162.0 ± 26.4196/216	U = 18,941.000, z = −3.306, *p* = 0.001^b^	170.0 ± 23.3237/277	162.8 ± 27.0 141/160	159.8 ± 24.855/56	χ^2^ 12.509, *p* = 0.002, df = 2^c^	TGA > TIA (z = −3.092, *p* = 0.006)^d^ TGA > CS (z = 2.475, *p* = 0.04)^d^
Diastolic blood pressure (DBP) on admission	92.2 ± 13.0 226/277	87.1 ± 17.3196/216	U = 17,355.000, z = −3.837, *p* < 0.001^b^	92.2 ± 13.0226/277	87.4 ± 17.6141/160	86.4 ± 16.555/56	χ^2^ 14.722, *p* = 0.001, df = 2^c^	TGA > TIA (z = −2.497, *p* = 0.038)^d^ TGA > CS (z = 3.486, *p* = 0.001)^d^
Hypertensive crisis on admission^f^	72.2% 171/237	57.7%113/196	χ^2^ = 9.992, *p* = 0.002^a^	72.2%171/237	60.3% 85/141	50.1%28/55	χ^2^ = 11.533, *p* = 0.003^a^	TGA (z = −3.161, *p* = 0.007)^d^

a *Chi square*.

b *Mann-Whitney-U-Test*.

c *Kruskal-Wallis test used as appropriate. Post-hoc p-values were adjusted via*.

d inverse Bonferroni (p_adj_ = p ^*^ k, k being the number of comparisons) in order to correct for multiple testing and alpha error accumulation. n.s., not significant.

e *CHA_2_DS_2_-VASc score on admission: for AIS-Patients, the current stroke event was not included, but a previous stroke event was considered.*

f *Definition of hypertensive crisis on admission: (1) in chronic hypertensive patients (= antihypertensive medication was required during hospital stay and at discharge), systolic blood pressure value > 179 mm Hg and/or diastolic blood pressure value > 109 mm Hg, and (2) in normotensive patients (no antihypertensive medication was required after day of admission and at discharge), systolic blood pressure value > 159 mm Hg and/or diastolic blood pressure value > 99 mm Hg*.

#### Vascular Risk Factors

**Figure 2:**
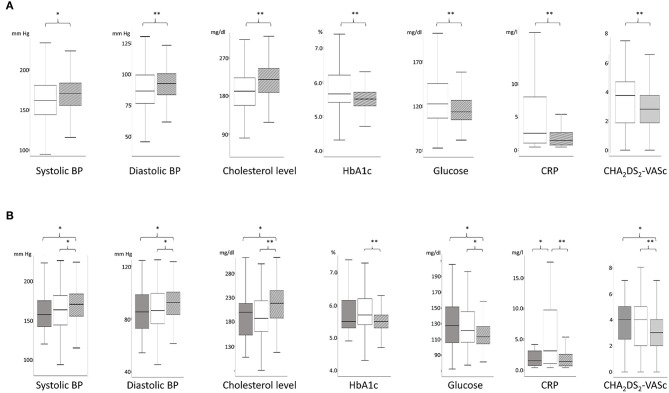
**(A)** Comparison of the parameters of the vascular risk profile between patients with stroke (AIS-patients, white) and TGA (TGA-patients, gray) using boxplots. ^*^p < 0.05; ^**^p < 0.001. In this figure, stroke patients include patients with transient ischemic attack (TIA) and completed stroke (CS). **(B)** Comparison of the parameters of the vascular risk profile between patients with TIA (TIA-patients, dark gray; left boxplots), completed stroke (CS-patients, white, middle boxplots), and TGA (TGA-patients, light gray, right boxplots). ^*^p < 0.05; ^**^p < 0.001.

Differences in vascular risk factors between TGA-patients and AIS-patients were analyzed using Mann-Whitney-U test due to non-normal distribution of the data. In the first analysis, all AIS-patients were included (*N* = 216), without differentiating between those with CS and TIA. When compared to AIS-patients, TGA-patients had significantly higher systolic blood pressure (SBP) and diastolic blood pressure (DBP), higher cholesterol levels, lower HbA1c as well as glucose levels, lower CRP values, and lower CHA_2_DS_2_-VASc scores (see Figure 2A). The mean values of the previously mentioned cardiovascular risk factors and the statistical differences between the groups are shown in Table 2. At discharge, TGA-patients had less frequently antihypertensive therapy compared to AIS-patients. TGA patients were significantly more likely to have hypertensive crises on admission compared to AIS patients (Table 2). The presence of cardiovascular risk factors is also shown in Table 2 including absolute and relative frequencies as well as statistical group differences. Patients with AIS were significantly more likely to have septal hypertrophy compared with TGA patients and were more likely to require antihypertensive medication at discharge.

### Secondary Analysis: TGA vs. CS vs. TIA

#### Demographic Characteristics

After confirming the differences between TGA-patients and AIS-patients, a more in-depth comparison of TGA-patients (*N* = 277), CS-patients (*N* = 160), and TIA-patients (*N* = 56) was conducted. Kruskal-Wallis tests revealed significant differences regarding age, with younger TGA-patients and TIA-patients (69.4 ± 14.7 years) and older CS-patients (71.1 ± 13.9 years). *Post-hoc* tests showed that only the age of TGA-patients and CS-patients differed significantly, while TIA-patients did not differ from either TGA-patients or AIS-patients. Statistically significant differences were also observed for gender, with a higher percentage of women in TGA-patients (61.0%) compared to CS-patients (45.0%) and TIA-patients (41.1%).

#### Vascular Risk Factors

Differences in vascular risk profile between patient groups were calculated using Kruskal-Wallis tests, due to non-normal distribution in at least one of the three groups as well as heteroscedasticity. When compared to both TIA-patients as well as CS-patients, TGA-patients had significantly higher SBP and DBP, higher cholesterol levels, lower glucose levels, and lower CHA_2_DS_2_-VASc scores (see Figure 2B and Table 2). HbA1c was higher exclusively in CS-patients compared to TGA-patients. CRP levels were higher in CS-patients compared to TGA-patients and TIA-patients. At discharge, antihypertensive therapy was less common in TGA-patients than in TIA-patients or CS-patients. In addition, TGA patients were less likely to have septal hypertrophy compared with CS and TIA patients. In contrast, TGA patients were significantly more likely to have hypertensive crises on admission compared to TGA-patients and TIA-patients (Table 2).

## Exploratory Analysis: Age-Dependent Comparisons Between TGA-Patients and AIS-Patients

Exploratory analyses were conducted in different age groups to assess possible age dependency of the effects, using the median as a cut-off value (≤69 vs. >69 years). Preliminary analyses showed that older patients had significantly lower DBP, lower cholesterol, and higher CHA_2_DS_2_-VASc scores compared to younger patients; the SBP did not differ. The age-dependent comparisons of TGA-patients and AIS-patients still revealed a higher DBP (younger patients: t = 92.337, *p* < 0.001; older patients: t = 82.029, *p* < 0.001), increased cholesterol levels (younger patients: t = 77.291, *p* < 0.001; older patients: U = 4,000.500, z = −4.313, *p* < 0.001) and elevated CHA_2_DS_2_-VASc scores (younger patients: U = 12,848.000, z = 9.539, *p* < 0.001; older patients: U = 11,977.000, z = 9.407, *p* < 0.001) in AIS-patients vs. TGA-patients, regardless of age. Hence, the differences between the cardiovascular risk factors could not be explained by the age differences between TGA-patients and AIS-patients.

## Exploratory Analysis: Gender-Dependent Comparisons Between TGA and All Stroke Patients

Gender-specific analyses were carried out to assess possible influences of different gender distributions on vascular risk factors in TGA-patients and AIS-patients. Cholesterol levels and CHA_2_DS_2_-VASc scores were significantly higher in women compared to men. Other vascular risk factors (SBP, DBP, HbA1c, glucose, CRP) did not differ between women and men. Both male TGA-patients (Student's *t*-test: t = 4.314, *p* < 0.001) and female TGA-patients (U = 5,252.000, z = −3.374, *p* = 0.001) had higher cholesterol levels compared to AIS-patients of the same gender. Analysis of CHA_2_DS_2_-VASc scores revealed that both male (U = 11,197.500, z = 9.423, *p* < 0.001), and female (U = 14,056.000, z = 10.254, *p* < 0.001) AIS-patients showed higher CHA_2_DS_2_-VASc scores compared to TGA-patients of the same gender. Therefore, the differences between AIS-patients and TGA-patients could not be explained by previous gender differences in the two groups.

## MR Imaging Abnormalities

A total of 251 TGA-patients underwent MRI. They were compared to 118 AIS-patients. Extent of the cerebral microangiopathy was evaluated using the Fazekas' score in available MRI images. No cerebral microangiopathy (Fazekas' score 0) was found in 35.1% of TGA-patients (25.4% of AIS-patients), mild microangiopathy (score 1) in 52.6% (28.8%), moderate microangiopathy (score 2) in 9.6% (22.9%), and severe microangiopathy (score 3) in 2.8% (22.9%). Distribution of microangiopathic lesions using Fazekas' score in TGA-patients and AIS-patients is displayed in Figure 1B. Using Mann-Whitney-U test, the degree of microangiopathy (ranked from 0 = none to 3 = severe) was significantly higher in AIS-patients compared to TGA-patients (U = 19,502.500, z = 5.257, *p* < 0.001). The effect size according to Cohen was r = 0.27 corresponding to a small effect with a trend toward medium effect size.

Supplementary analysis using the Kruskal-Wallis test (χ^2^ 30.339, *p* < 0.001, df = 2) between the three groups (TGA, CS, TIA) showed that this effect was due to higher severity of microangiopathy in CS-patients compared to TGA-patients (z = −5.402, *p* < 0.001). The extent of microangiopathy in TIA-patients compared to TGA-patients (z = 2.011, *p* = 0.133) and CS-patients (z = −1.644, *p* = 0.301) did not show significant differences.

## Hippocampal Lesions in TGA-Patients

Of 251 TGA-patients undergoing MRI scan, 132 (52.6%) showed typical hippocampal DWI lesions. In 29 patients (11.6%), bilateral DWI lesions were observed. In TGA-patients with one-sided DWI lesion, the sides were evenly distributed (left *N* = 51, 20.3%, and right *N* = 52, 20.7%). The frequency of hippocampal DWI lesions in TGA-patients is displayed in Figure 1C. Gender did not differ between TGA-patients with (62.1% female) and without (61.2% female) DWI lesion (χ^2^ 0.000, *p* = 0.992).

Analyses of age and cardiovascular risk factors in TGA-patients with and without hippocampal lesion were carried out. Non-parametric tests revealed that TGA-patients without DWI lesion were older (median age 68 years) than TGA-patients with DWI lesion (median age 66 years) (U = 6,662.000, z = −2.077, *p* = 0.038) and had higher CHA_2_DS_2_-VASc scores (mean 3.1 ± 1.6 vs. 2.6 ± 1.5, U = 6,495.500, z = −2.411, *p* = 0.016). HbA1c, glucose level, and CRP level did not differ significantly between both groups. Correspondingly, normally distributed parameters (SBP, DBP, cholesterol level) did not provide statistically significant results either. Interestingly, patients without DWI lesion more frequently showed septal hypertrophy (>11 mm) on transthoracic echocardiography (40.0 vs. 22.9%, χ2 = 4.082, *p* = 0.043) (see Figure 1D). However, according to a predefined increased septal thickness (women > 9 mm, men > 10 mm) (24), the measured values did not reach significance. Neither the presence of DWI lesions nor the severity of cerebral microangiopathy were significantly associated with reduced left ventricular ejection fraction <50% in transthoracic echocardiography.

## Discussion

TGA-patients in our collective were significantly younger and predominantly female when compared to AIS-patients. 90.6% of TGA-patients received an MRI, 53% of those undergoing MRI scan displayed a hippocampal DWI lesion. TGA-patients without DWI lesions were older and showed higher CHA_2_DS_2_-VASc scores compared to TGA-patients with DWI lesions.

The extent of cerebral microangiopathy in TGA-patients was lower compared to AIS-patients. TGA-patients showed less frequently septal hypertrophy in transthoracic echocardiography as a potential sign of chronic hypertensive heart disease (27) and required less frequently antihypertensive medication at discharge compared to AIS-patients. In contrast, TGA-patients showed a significantly higher blood pressure at symptom onset. Cholesterol levels in these patients were also higher compared to AIS-patients. As expected, TGA-patients displayed lower HbA1c levels, blood glucose levels, and CHA_2_DS_2_-VASc scores, whereas, CS-patients initially had increased CRP levels compared to TIA-patients and TGA-patients.

## Hypertension and Cerebral Microangiopathy

The most intriguing and important finding of our study was the hypertensively dysregulated blood pressure at symptom onset, being significantly higher compared to CS-patients and TIA-patients. Yet, at discharge TGA-patients had less frequently antihypertensive therapy compared to TIA-patients and CS-patients. Antihypertensive medication at discharge is used as a marker for chronic hypertension in this context. Septal hypertrophy as another possible marker of chronic hypertension also persists less frequently in TGA patients. These observations suggest an acute dysregulation of blood pressure in the acute phase of the disease. TGA-patients were obviously not adapted to high blood pressure episodes, which may act as a possible trigger for metabolic stress in the vulnerable hippocampal region causing TGA. The CA1 sector of the hippocampal cornu ammonis is known to show a selective vulnerability to metabolic and oxidative stress caused by hypoxaemia, β-amyloid-induced neurotoxicity, and ischaemia mediated by glutamate overload and calcium influx (9, 28). Several studies have described emotional, physical, and behavioral stress situations preceding the onset of TGA (9).

The brain is protected from extremes of blood pressure by an autoregulation system that ensures constant perfusion over a wide range of systemic pressures. In normotensive individuals, cerebral blood flow remains unchanged between mean blood pressures of ~60 and 150 mm Hg. At pressures above the upper limit of autoregulation, hypertensive encephalopathy may occur (29). Our data present acute hypertensive peaks as a possible trigger for TGA, especially in patients not adapted to hypertension. The bilateral occurrence of DWI lesions in a number of patients further supports this hypothesis (as opposed to microembolic infarcts). This theory is fundamentally supported by the observation that AIS-patients in our study had a significantly higher incidence of microangiopathic lesions, which in turn is indicative of cerebrovascular risk factors, such as chronic hypertension (30). Patients with chronic hypertension suffer from hypertrophy of the arterioles, which reduces the transmission of blood pressure to the capillary region. Thus, hypertensive emergencies occur only at higher blood pressure levels. However, the acute phase of TGA may be considered as a form of hypertensive encephalopathy that goes beyond an isolated hypertensive urgency due to the neurological symptoms.

The observation of higher blood pressure values in TIA-patients compared to TGA-patients has been described before (21, 31) but was never assessed systematically in the acute phase of the syndrome in a representative patient cohort. Several studies investigated the association with chronic hypertension (16, 21, 31). In contrast, in our study we analyzed blood pressure values in the initial phase after symptom onset. The only study which reported elevated SBP values in the acute phase was by Nedelmann et al. (32). In this study, 21 of 22 patients with TGA showed elevated mean pressure 3 h after onset of symptoms (180.9/98.3 mmHg), and one-third of patients had SPB values above 200 mmHg. In our study we can systematically demonstrate a relevant influence of blood pressure dysregulation in the acute phase of TGA in a larger cohort and in comparison to stroke patients.

Regarding microangiopathy, the study by Enzinger et al. showed no significant difference in the extent of microangiopathy between TGA-patients and HC (16). Another study showed a similar prevalence of cerebral microangiopathy compared to our study (33).

## Hippocampal DWI Lesion

MRI reveals typical DWI lesions with corresponding T2-lesions in the CA1 region of the hippocampus 24–72 h after TGA, which are detectable for 10–14 days (8). An analysis of 17 studies showed that in more than half of TGA-patients DWI lesions in the hippocampus are detectable if appropriate imaging techniques are applied (7). These lesions are more often unilateral but can also occur bilaterally. If no hippocampal lesions are encountered, first of all technical aspects (timing, acquisition protocol) should be considered and carefully be reviewed. Frequently, MRI scan is performed early to disclose AIS. In these cases, a repeated MRI scan within the appropriate time window for proof of TGA lesions might be indicated. Yet, absence of characteristic DWI lesions does not rule out TGA, i.e., clinical TGA criteria do not require a confirmation by MRI. In our TGA population, MRI was not performed exclusively to detect typical hippocampal DWI lesions, but rather frequently to disclose a stroke.

In our study, TGA-patients without DWI lesions were older and had a higher CHA_2_DS_2_-VASc score. Interestingly, patients without DWI lesions showed more septal hypertrophy (>11 mm) in transthoracic echocardiography as a potential sign of chronic hypertensive heart disease. However, according to a predefined increased septal thickness (women > 9 mm, men > 10 mm) (24), the measured values did not reach significance, so this cannot be considered a robust finding but merely an indication of possible chronic hypertension. Furthermore, septal hypertrophy is not specific evidence of hypertensive heart disease and may have other causes (27).

In previous studies, the vascular risk profile of TGA-patients and concomitant changes on brain MRI did not show significant differences between DWI-positive and DWI-negative subjects (16). Winbeck et al. (7) described that TGA-patients with DWI lesions had a more pronounced vascular risk profile and were significantly more likely to have carotid atherosclerosis than DWI-negative TGA-patients.

In contrast, our data suggest that the detection of DWI lesions could also be a sign of lack of adaptation to the trigger hypertensive dysregulation. The higher CHA_2_DS_2_-VASc score and more frequently detected septal hypertrophy are signs of a possible adaptation to hypertension.

## Other Risk Factors

Our study suggests a gender distribution of 61% women and 39% men. Although, other studies reported also a female predominance (3, 14, 18, 34), a meta-analysis revealed no significant gender difference (46.4% men and 53.6% women; χ^2^ = 0.48, *p* = 0.49) between the 1,333 patients collected from 52 published case series (*n* = 91) and 34 published group studies (*n* = 1,171) (34). More recent studies published after this meta-analysis also reported a female predominance (14, 33). A possible explanation could be the frequent diagnosis of stroke mimics in women despite similar symptoms compared to men (35).

The current study showed a significant age difference between TGA-patients and AIS-patients. The mean age of our TGA population was in line with the mean age of many other reported study populations (14, 20, 21, 31, 33, 36). Furthermore, the age of our AIS-patients was consistent with other large stroke cohorts (37, 38) in previous studies.

The current study showed a higher prevalence of hyperlipidemia on admission in TGA-patients compared to AIS-patients. This observation was also reported in a previous study by Jang et al. (21). Other studies demonstrated lower lipid levels in TGA-patients compared to TIA-patients (18). In the reported study (21), the proportion of TGA-patients who had previously received lipid-lowering therapy was significantly lower than that of TIA-patients or HC. The authors concluded that the number of patients first diagnosed with hyperlipidemia was higher when evaluated for TGA than those of TIA-patients or HC.

A decreased prevalence of diabetes mellitus in TGA-patients compared to TIA-patients was previously reported (7, 18, 20, 21), which is in line with our findings, where blood glucose levels were lower in TGA-patients compared to AIS-patients. These figures might indicate a less pronounced cardiovascular risk profile.

Regarding inflammation, elevated levels of C-reactive protein (CRP) are present among patients at risk for first-ever myocardial infarction and stroke (39). Furthermore, elevated CRP levels independently predict the risk of future stroke and transient ischemic attack in the elderly (40). To our knowledge, systematic investigation of CRP levels in TGA-patients has not yet been published. Our study showed significantly lower CRP levels in TGA-patients.

An age-matched analysis by Lauria et al. found that TGA-patients had a significantly lower prevalence of atrial fibrillation than TIA-patients (20). These data are consistent with our observation that TGA-patients have lower CHA_2_DS_2_-VASc scores.

Due to the retrospective study design, the exact time of symptom onset and the resulting time interval between symptom onset and the MRI examination could not be clearly ascertained in all cases. Furthermore, in many cases, the retrospective study design did not allow tracking of prior lipid-lowering medication. Thus, the evidence of a higher prevalence of hypercholesterolemia may be related to lipid-lowering therapy in stroke patients.

## Conclusion

In conclusion, our data support the observation of significant differences in the vascular risk profile between TGA-patients and AIS-patients. TGA-patients are younger, more often female, have higher prevalence of hyperlipidemia, lower blood glucose levels, lower CRP levels, and lower CHA_2_DS_2_-VASc scores compared to AIS-patients. TGA-patients are less likely to have chronic hypertension, which is reflected in lower levels of consecutive hypertensive disorders such as the extent of cerebral microangiopathy and septal hypertrophy. However, TGA-patients have higher blood pressure values on admission compared to TIA-patients as well as CS-patients. This may be explained by the fact that patients with chronic hypertension have the opportunity to get adapted to hypertensive blood pressure values, but carry in the long run a higher risk of secondary diseases such as heart attack and stroke. Patients without chronic hypertension seem to be more sensitive to hypertensive peaks and are more likely to suffer TGA as a result of these acute dysregulation causing metabolic stress in the vulnerable hippocampal CA1 sector.

Our study design do not allow us to prove a causal relationship, but the observation of an increased risk of TGA during physical exercise, Valsalva maneuvers, as well as coitus support the observation that blood pressure peaks may play an important role in the pathophysiology of TGA.

## Code Availability

Statistical analysis of the data was carried out using the Statistical Package for the Social Sciences (SPSS) version 25.0.0.2 (IBM, 2018).

## Data Availability Statement

The raw data supporting the conclusions of this article are available from the corresponding author upon reasonable request.

## Ethics Statement

Our study complied with the guidelines for human studies and was conducted ethically in accordance with the Declaration on Ethics of the World Medical Association of Helsinki. The study was approved by the local ethics committee of Muenster (file reference: 2021-288-f-S).

## Author Contributions

AR, AB, and W-RS designed the study and drafted the manuscript. AR, AB, and JP performed data acquisition. AR and AF performed statictical analysis. RK and FW performed MRI analysis. FZ, IG, RK, and CB revised the manuscript. All were involved in data evaluation and discussions. All authors contributed to the article and approved the submitted version.

## Conflict of Interest

CB receives research support from the Deutsche Forschungsgemeinschaft (German Research Council, Bonn, Germany) and Gerd-Altenhof-Stiftung (Deutsches Stiftungs-Zentrum, Essen, Germany). The remaining authors declare that the research was conducted in the absence of any commercial or financial relationships that could be construed as a potential conflict of interest.

## Abbreviations

DWI: diffusion-weighted imaging
TGA-patients: patients with transient global amnesia
AIS-patients: patients with acute ischemic stroke (completed stroke as well as TIA)
TIA-patients: patients with transient ischemic attack
CS-patients: patients with completed stroke
HC: healthy controls
SBP: systolic blood pressure
DBP: diastolic blood pressure.

## References

[1] Courjon J Guyotat J. Amnesic strokes. J Med Lyon. (1956) 37:697–701. 13377072

[2] Lewis SL. Aetiology of transient global amnesia. Lancet. (1998) 352:397–9. 10.1016/S0140-6736(98)01442-1 9717945

[3] Lauria G Gentile M Fassetta G Casetta I Caneve G. Incidence of transient global amnesia in the Belluno province, Italy: 1985 through (1995). Acta Neurol Scand. (1997) 95:303–10. 10.1111/j.1600-0404.1997.tb00215.x 9188907

[4] Koski KJ Marttila RJ. Transient global amnesia: incidence in an urban population. Acta Neurol Scand. (2009) 81:358–60. 10.1111/j.1600-0404.1990.tb01571.x 2360405

[5] Kritchevsky M Squire RL. Transient global amnesia: evidence for extensive, temporally graded retrograde amnesia. Neurology. (1989) 39:213–8. 10.1212/WNL.39.2.213 2915792

[6] Sedlaczek OL Hirsch JG Grips E Peters CNA Gass A Wöhrle J. Detection of delayed focal MR changes in the lateral hippocampus in transient global amnesia. Neurology. (2004) 62:2165–70. 10.1212/01.WNL.0000130504.88404.C9 15210876

[7] Winbeck K Etgen T Von Einsiedel HG Rôttinger M Sander D. DWI in transient global amnesia and TIA: Proposal for an ischaemic origin of TGA. J Neurol Neurosurg Psychiatry. (2005) 76:438–41. 10.1136/jnnp.2004.042432 15716545 PMC1739538

[8] Bartsch T Alfke K Stingele R Rohr A Freitag-Wolf S Jansen O. Selective affection of hippocampal CA-1 neurons in patients with transient global amnesia without long-term sequelae. Brain. (2006) 129:2874–84. 10.1093/brain/awl248 17003071

[9] Bartsch T Deuschl G. Transient global amnesia: functional anatomy and clinical implications. Lancet Neurol. (2010) 9:205–14. 10.1016/S1474-4422(09)70344-8 20129169

[10] Lee HY Kim JH Weon YC Lee JS Kim SY Youn SW. Diffusion-weighted imaging in transient global amnesia exposes the CA1 region of the hippocampus. Neuroradiology. (2007) 49:481–7. 10.1007/s00234-007-0213-5 17522744

[11] Szabo K Hoyer C Caplan LR Grassl R Griebe M Ebert A. Diffusion-weighted MRI in transient global amnesia and its diagnostic implications. Neurology. (2020) 95:E206–12. 10.1212/WNL.0000000000009783 32532848

[12] Uttner I Prexl S Freund W Unrath A Bengel D Huber R. Long-term outcome in transient global amnesia patients with and without focal hyperintensities in the CA1 region of the hippocampus. Eur Neurol. (2012) 67:155–60. 10.1159/000334735 22261698

[13] Buhr J Evers S Husstedt IW Frese A. Event related potentials in patients with transient global amnesia - a prospective controlled study. J Neurol Sci. (2013) 325:57–60. 10.1016/j.jns.2012.11.017 23260318

[14] Romoli M Tuna MA McGurgan I Li L Giannandrea D Eusebi P. Long-term risk of stroke after transient global amnesia in two prospective cohorts. Stroke. (2019) 50:2555–7. 10.1161/STROKEAHA.119.025720 31284848

[15] Zorzon M Antonutti L Mase G Biasutti E Vitrani B Cazzato G. Transient global amnesia and transient ischemic attack: natural history, vascular risk factors, and associated conditions. Stroke. (1995) 26:1536–42. 10.1161/01.STR.26.9.1536 7660394

[16] Enzinger C Thimary F Kapeller P Ropele S Schmidt R Ebner F. Transient global amnesia: diffusion-weighted imaging lesions and cerebrovascular disease. Stroke. (2008) 39:2219–25. 10.1161/STROKEAHA.107.508655 18583561

[17] Mangla A Navi BB Layton K Kamel H. Transient global amnesia and the risk of ischemic stroke. Stroke. (2014) 45:389–93. 10.1161/STROKEAHA.113.003916 24309586 PMC3946840

[18] Melo TP Ferro JM Ferro H. Transient global amnesia: a case control study. Brain. (1992) 115:261–70. 10.1093/brain/115.1.261 1559158

[19] Pantoni L Bertini E Lamassa M Pracucci G Inzitari D. Clinical features, risk factors, and prognosis in transient global amnesia: a follow-up study. Eur J Neurol. (2005) 12:350–6. 10.1111/j.1468-1331.2004.00982.x 15804264

[20] Lauria G Gentile M Fassetta G Casetta I Caneve G. Transient global amnesia and transient ischemic attack: a community- based case-control study. Acta Neurol Scand. (1998) 97:381–5. 10.1111/j.1600-0404.1998.tb05970.x 9669471

[21] Jang JW Park SY Hong JH Park YH Kim JE Kim S. Different risk factor profiles between transient global amnesia and transient ischemic attack: a large case-control study. Eur Neurol. (2014) 71:19–24. 10.1159/000354023 24281363

[22] Romero JR Mercado M Beiser AS Pikula A Seshadri S Kelly-Hayes M. Transient global amnesia and neurological events: the framingham heart study. Front Neurol. (2013) 4:47. 10.3389/fneur.2013.00047 23675365 PMC3653124

[23] Hodges JR Warlow CP. Syndromes of transient amnesia: towards a classification. A study of 153 cases. J Neurol Neurosurg Psychiatry. (1990) 53:834–43. 10.1136/jnnp.53.10.834 2266362 PMC488242

[24] Lang RM Badano LP Victor MA Afilalo J Armstrong A Ernande L. Recommendations for cardiac chamber quantification by echocardiography in adults: an update from the American Society of Echocardiography and the European Association of Cardiovascular Imaging. J Am Soc Echocardiogr. (2015) 28:1–39.e14. 10.1016/j.echo.2014.10.003 25559473

[25] Cantone M Lanza G Puglisi V Vinciguerra L Mandelli J Fisicaro F. Hypertensive crisis in acute cerebrovascular diseases presenting at the emergency department: a narrative review. Brain Sci. (2021) 11:1–28. 10.3390/brainsci11010070 33430236 PMC7825668

[26] Vaughan CJ Delanty N. Hypertensive emergencies. Lancet. (2000) 356:411–7. 10.1016/S0140-6736(00)02539-3 10972386

[27] Loncaric F Nunno L Mimbrero M Marciniak M Fernandes JF Tirapu L. Basal ventricular septal hypertrophy in systemic hypertension. Am J Cardiol. (2020) 125:1339–46. 10.1016/j.amjcard.2020.01.045 32164912

[28] Kosuge Y Imai T Kawaguchi M Kihara T Ishige K Ito Y. Subregion-specific vulnerability to endoplasmic reticulum stress-induced neurotoxicity in rat hippocampal neurons. Neurochem Int. (2008) 52:1204–11. 10.1016/j.neuint.2007.12.010 18280615

[29] Lamy C Mas JL. Hypertensive encephalopathy. In: Stroke: Pathophysiology, Diagnosis, and Management. Amsterdam: Elsevier Inc. (2016). p. 640–7. 10.1016/B978-0-323-29544-4.00038-4

[30] de Leeuw FE de Groot JC Oudkerk M Witteman JCM Hofman A van Gijn J. Hypertension and cerebral white matter lesions in a prospective cohort study. Brain. (2002) 125:765–72. 10.1093/brain/125.4.765 11912110

[31] Himeno T Kuriyama M Takemaru M Kanaya Y Shiga Y Takeshima S. Vascular risk factors and internal jugular venous flow in transient global amnesia: a study of 165 Japanese patients. J Stroke Cerebrovasc Dis. (2017) 26:2272–8. 10.1016/j.jstrokecerebrovasdis.2017.05.010 28669658

[32] Nedelmann M Kaps M. Elevated blood pressure as a prominent finding in patients with transient global amnesia. Eur J Neurol. (2007) 14:e22. 10.1111/j.1468-1331.2007.01846.x 17594310

[33] Waliszewska-Prosol M Nowakowska-Kotas M Bladowska J Papier P Budrewicz S Pokryszko-Dragan A. Transient global amnesia - risk factors and putative background. Neurol India. (2020) 68:624. 10.4103/0028-3886.288979 32643675

[34] Quinette P Guillery-Girard B Dayan J La Sayette VD Marquis S Viader F. What does transient global amnesia really mean? Review of the literature and thorough study of 142 cases. Brain. (2006) 129:1640–58. 10.1093/brain/awl105 16670178

[35] Yu AYX Penn AM Lesperance ML Croteau NS Balshaw RF Votova K. Sex differences in presentation and outcome after an acute transient or minor neurologic event. JAMA Neurol. (2019) 76:962–8. 10.1001/jamaneurol.2019.1305 31114842 PMC6537759

[36] Arena JE Brown RD Mandrekar J Rabinstein AA. Long-term outcome in patients with transient global amnesia: a population-based study. Mayo Clin Proc. (2017) 92:399–405. 10.1016/j.mayocp.2016.11.015 28185658 PMC5682935

[37] Kissela BM Khoury JC Alwell K Moomaw CJ Woo D Adeoye O. Age at stroke: temporal trends in stroke incidence in a large, biracial population. Neurology. (2012) 79:1781–7. 10.1212/WNL.0b013e318270401d 23054237 PMC3475622

[38] Misselwitz B Grau A Berger K Bruder I Burmeister C Hermanek P. Quality of care of acute ischemic stroke in Germany (2018). Nervenarzt. (2020) 91:484–92. 10.1007/s00115-020-00908-x 32350547

[39] Winbeck K Poppert H Etgen T Conrad B Sander D. Prognostic relevance of early serial C-reactive protein measurements after first ischemic stroke. Stroke. (2002) 33:2459–64. 10.1161/01.STR.0000029828.51413.82 12364738

[40] Rost NS Wolf PA Kase CS Kelly-Hayes M Silbershatz H Massaro JM. Plasma concentration of c-reactive protein and risk of ischemic stroke and transient ischemic attack: the Framingham Study. Stroke. (2001) 32:2575–9. 10.1161/hs1101.098151 11692019

